# Effect of single-visit full-mouth non-surgical therapy and risk factor analysis on long-term periodontal treatment outcomes: A retrospective study

**DOI:** 10.1007/s00784-025-06405-2

**Published:** 2025-06-05

**Authors:** Obada Mandil, Abdusalam Alrmali, Khushboo Kalani, Bidisha Ray, Parth Ghataliya, Shahad Alhazmi, Robert A. Levine, Muhammad H.A. Saleh, Hom-Lay Wang

**Affiliations:** 1 Department of Periodontics and Oral Medicine, School of Dentistry, University of Michigan, Ann Arbor, MI USA; 2 Department of Periodontics, School of Dental Medicine, Case Western Reserve University, Cleveland, OH USA; 3 Department of Oral Medicine, Oral Pathology, Oral and Maxillofacial Surgery, University of Tripoli School of Dentistry, Tripoli, Libya; 4 Department of Restorative Dentistry, School of Dentistry, University of Michigan, Ann Arbor, MI USA; 5 Department of Periodontics, School of Dental Medicine, University of Pittsburgh, Pittsburgh, PA USA

**Keywords:** Periodontitis, Supportive periodontal therapy, Tooth loss, Smoking

## Abstract

**Objectives:**

This study aimed to evaluate the long-term effectiveness of single-visit full-mouth non-surgical therapy in managing patients with progressive periodontitis during supportive periodontal therapy (SPT), with tooth loss due to periodontitis (TLP) as the primary outcome. Secondary objectives included assessing changes in probing depth (PD) and identifying risk factors associated with TLP.

**Materials and methods:**

A retrospective analysis was conducted using patient records from the University of Michigan School of Dentistry. Included were 283 patients (mean age: 53.2 years) with periodontal breakdown during SPT (PiKS) who underwent single-visit full-mouth ultrasonic instrumentation. The primary outcome was TLP over a mean follow-up of 19.4 years. Secondary outcomes included changes in PD (≥ 5 mm and ≥ 6 mm) and identification of risk factors for tooth loss. Statistical analyses used multilevel binary logistic regression with generalized estimating equations (GEE) and linear regression models.

**Results:**

Among 283 patients (mean age: 53.2 years), the mean TLP was 0.9 teeth per patient over a mean follow-up of 19.4 years. Factors significantly associated with TLP included diabetes (OR = 2.41; *p* = 0.013), current smoking (OR = 2.13; *p* = 0.025), higher periodontitis Grades B and C (OR = 3.31; *p* = 0.001), and Stages III-IV (OR = 8.67; *p* = 0.001). Baseline pocket depths (PD) ≥ 5 mm (OR = 1.13; *p* = 0.002) and ≥ 6 mm (OR = 1.29; *p* = 0.001) were also associated with higher TLP. Each additional annual SPT visit reduced the TLP risk by half (OR = 0.50; *p* = 0.003). PD ≥ 5 mm showed minimal increase change (0.16; *p* = 0.02), while depths ≥ 6 mm actually decreased (-0.10; *p* = 0.01).

**Conclusion:**

Single-visit full-mouth non-surgical therapy is effective for long-term management of PiKS, with low tooth loss rates. Diabetes, smoking, advanced periodontitis stage/grade, and deeper baseline pockets are key predictors of TLP. Frequent SPT visits significantly mitigate tooth loss risk.

**Clinical relevance:**

During (SPT) appointments, prevalent practice involves the full-mouth instrumentation of persistent periodontal pockets to disrupt microbial populations and consequently reduce the inflammatory response responsible for disease progression. This study investigates the efficacy of single-visit full-mouth instrumentation as a potential alternative to standard practices in controlling periodontal disease during SPT, which have potential benefits for patients, including reduced treatment time, improved adherence to maintenance therapy, and comprehensive management of periodontal disease. By minimizing the number of visits, this approach may enhance patient compliance while reducing the risk of reinfection from untreated sites between sessions, ultimately contributing to better long-term periodontal stability. Factors such as deeper baseline probing depths and crucial risk elements for TLP—including diabetes, smoking, and advanced stages (III-IV) and grades (B and C) of periodontitis, can play a role in TLP. Additionally, this study provides valuable insights into the customization of more intensive interventions for patients at higher risk. Our findings highlight the importance of frequent SPT visits, with each additional annual visit halving the risk of TLP. The observed reduction in PD of ≥ 6 mm following treatment indicates the significant potential of comprehensive debridement for enhancing long-term periodontal stability. This evidence supports the implementation of tailored, intensive SPT schedules, particularly for patients identified as having higher risks, thereby contributing to improved clinical outcomes in periodontal disease management.

**Supplementary Information:**

The online version contains supplementary material available at 10.1007/s00784-025-06405-2.

## Background and objectives

The primary treatment of periodontitis involves removing the dental biofilm and calcified deposits from the tooth surface, supplemented by effective management of supra- and sub-gingival biofilms, with well-documented efficacy [1, 2]. However, this approach is time-intensive, requires operator expertise, and can cause patient discomfort [3]. A straightforward matrix combining periodontitis Stage and Grade aids in outlining individual cases, indicating severity and management complexity, along with the risk of progression and systemic implications [4, 5].

Simple scoring systems were designed to evaluate periodontally affected teeth and can be easily calculated using examination data [6]. It demonstrated outstanding predictive ability for tooth loss due to periodontitis TLP in both the anterior and posterior regions. Implementing such or a comparable prognosis system is highly recommended for determining periodontal tooth prognosis [7, 8].

According to the American Academy of Periodontology (AAP) and recent European Federation of Periodontology (EFP) guidelines, periodontal maintenance therapy-also referred to as supportive periodontal therapy (SPT) or supportive periodontal care (SPC)-aims to prevent the recurrence of periodontal disease by minimizing the risks of tooth loss and further attachment loss. This is achieved through regular clinical assessments, risk evaluation, and thorough removal of biofilm and calculus, as well as ongoing patient education and reinforcement of oral hygiene practices. The latest evidence-based guidelines recommend that SPT is essential for maintaining periodontal stability following active therapy and should be tailored to individual risk profiles, with maintenance intervals often set at three to four months [9]. One common practice during SPT appointments is performing full-mouth debridement of persistent periodontal pockets to disrupt any microbial populations, thus reducing the inflammatory response driving disease progression. SPT enhances periodontal health and allows swift identification and intervention in cases of periodontal disease recurrence or progression [10–12]. Long-term studies consistently report low TLP rates of only 2–5% among patients initially treated for chronic periodontitis over 5 to 10 years, supporting the effectiveness of SPT [13–15].

Additionally, the understanding that deliberate removal of tooth structure is not always necessary for periodontal tissue healing, as effective removal of entire bacterial biofilm from the root surface, from the root surface can be achieved, supports the development of complete-mouth ultrasonic debridement protocols [16]. Initially proposed by Smart et al. [17], this conservative protocol involves intersecting strokes and gentle pressure applied for a finite period to establish a biocompatible root surface [18].

According to Iorio-Siciliano et al. (2024), non-surgical periodontal therapy, including scaling and root planing, remains a fundamental approach for periodontal treatment. It suggests that adjunctive therapies, such as locally delivered antibiotics and host-modulating agents, may enhance clinical outcomes, including PD reduction and CAL gain [19].

Despite indications suggesting the potential effectiveness of comprehensive ultrasonic debridement in treating severe chronic periodontitis, there is a lack of information on its repercussions– such as bacterial reduction, host immune-inflammatory response, and clinical outcomes– when performed within a maximum timeframe of 45 min [20]. This gap extends to understanding its impact on periodontopathogens quantities and the resulting host immune-inflammatory response [21, 22].

To address this gap, the present study aimed to evaluate the impact of a single-visit full-mouth instrumentation treatment on halting the progression of periodontitis in patients with breaking down sites during SPT (PiKS). Hence, the primary outcome of this study was the number of TLP over the follow-up period. Secondary outcomes included changes in periodontal PD ≥ 5 mm and ≥ 6 mm, the impact of diabetes, smoking, and periodontitis severity on TLP, the effect of single-visit full-mouth instrumentation on periodontal stability, and the protective role of SPT in reducing TLP risk.

## Materials and methods

### Study population

Data were extracted from electronic patient charts of individuals undergoing periodontal treatment at the University of Michigan School of Dentistry between January 2001 and January 2021. The study adhered to the principles outlined in the Helsinki Declaration. Ethical approval was granted by the University of Michigan School Institutional Review Board (ID: HUM00157260) and Amendment eResearch (ID: Ame00122603).

#### Inclusion criteria

1. Patients diagnosed with periodontitis, who received active periodontal therapy (APT), and who subsequently enrolled in an SPT program (To be diagnosed with periodontal breakdown during supportive periodontal therapy (PiKS), patients must present with at least two non-adjacent breaking-down sites (sites with PD of ≥ 5 mm with or without bleeding) [23] with only single-visit full-mouth instrumentation performed to halt the breaking-down sites rather than scaling and root planing (SRP)).
2. Patients needed baseline dental, periodontal, and self-reported medical records, with tooth extractions conducted at the University of Michigan School of Dentistry.

#### Exclusion criteria

1. Patients treated outside the University of Michigan.
2. Patients with SPT failure for one year or more during the study period.

### Single-visit full-mouth instrumentation

After a diagnosis of PiKS was established (baseline examination, T0), all patients received non-surgical full-mouth periodontal instrumentation using ultrasonic cleaning tips during a single visit. Procedures were accomplished within 1–2-hour visits without local anesthesia. Patients received care from dental students, hygienists, and periodontics residents. All patients also received comprehensive and oral hygiene instructions. After completing active periodontal treatment, patients were re-evaluated and re-enrolled in the SPT program at the same institution. Patients after that attended SPT sessions at least once annually. Data collected at baseline (T0) were compared to data from the latest SPT visit (T1).

### Data collection

Electronic and physical records of eligible patients were assessed by two examiners (BR, PG). At the patient level, characteristics collected at baseline (T0) encompassed age, sex, history of diabetes mellitus (HbA1c), and smoking status. Smoking status was classified into five groups: non-smokers, former light smokers (< 10 cigarettes per day), former heavy smokers (> 10 cigarettes per day), current light smokers (< 10 cigarettes per day), and current heavy smokers (> 10 cigarettes per day). At the last visit (T1), follow-up duration and the count of SPT visits were calculated. Clinical and radiographic data were gathered at the tooth level at T0 and T1, excluding third molars. Clinical parameters, including PD, were assessed at six sites per tooth and overall TLP.

### Case definition

#### Periodontitis, staging and grading

Patients were classified according to the 2018 World Workshop Classification into periodontitis Stages (I, II, III, and IV) and Grades (A, B, and C) [24]. A single investigator (OM) carried out the individual case classification.

#### Pocket closure

Pocket closure was defined as a PD of ≤ 4 mm with or without bleeding on probing (BOP). The prevalence of closed and open pockets was assessed at baseline and the last follow-up visit.

### Statistical analysis

Binary outcomes were analyzed using multi-level binary logistic regression with generalized estimation equations (GEE). Raw odds ratios and 95% confidence intervals were derived from Wald’s Chi2 statistic. Multiple models were created to account for potential confounding factors. Quantitative outcomes, such as PD progression and total costs, were subjected to linear regression models with GEE equations to address within-subject tooth dependence. Beta coefficients and 95% confidence intervals were reported. Significance was set at 5% (α = 0.05).

## Results

### Relevant descriptive

#### Demographic characteristics

The study included 283 patients diagnosed with periodontitis who underwent single-visit full-mouth instrumentation. Patients were classified as 58 (20.5%) patients with Stage I, 195 (68.9%) patients with Stage II, 27 (9.5%) patients with Stage III, and 3 (1.1%) patients with Stage IV. For grading, they were classified as 207 (73.1%) patients with Grade A, 72 (25.4%) patients with Grade B, and 4 (1.4%) patients with Grade C. Of these patients, 109 (38.5%) were male and 174 (61.5%) were female. The mean age of the patients was 53.2 ± 12.6 years, ranging from 18 to 88 years. Patients were followed for an average duration of 19.4 ± 6.9 years, with a range of 0.2 to 26.3 years from treatment initiation (T0) to the final follow-up (T1). (Table 1) includes an analysis of the patient’s demographic features and site characteristics.

**Table 1:** Demographic table showing patient and site characteristics

Characteristic	*N*	%
Total Patients	283	100.0
Gender		
Male	109	38.5
Female	174	61.5
Age (years)		
≤ 45	76	26.9
46–55	90	31.8
56–65	69	24.4
> 65	48	17.0
Diabetes		
No	242	85.5
Yes	41	14.5
Smoking Status		
Non-smoker	201	71.0
Former smoker (< 10 c/d)	28	9.9
Former smoker (> 10 c/d)	5	1.8
Current smoker (< 10 c/d)	43	15.2
Current smoker (> 10 c/d)	6	2.1
Periodontitis Stage		
I	58	20.5
II	195	68.9
III	27	9.5
IV	3	1.1
Periodontitis Grade		
A	207	73.1
B	72	25.4
C	4	1.4
Follow-up (years)		
Mean ± SD (Range)	19.4 ± 4.9 (2–22.3)	

Abbreviations: c/d = cigarettes per day; SD = standard deviation

**Fig. 1:**
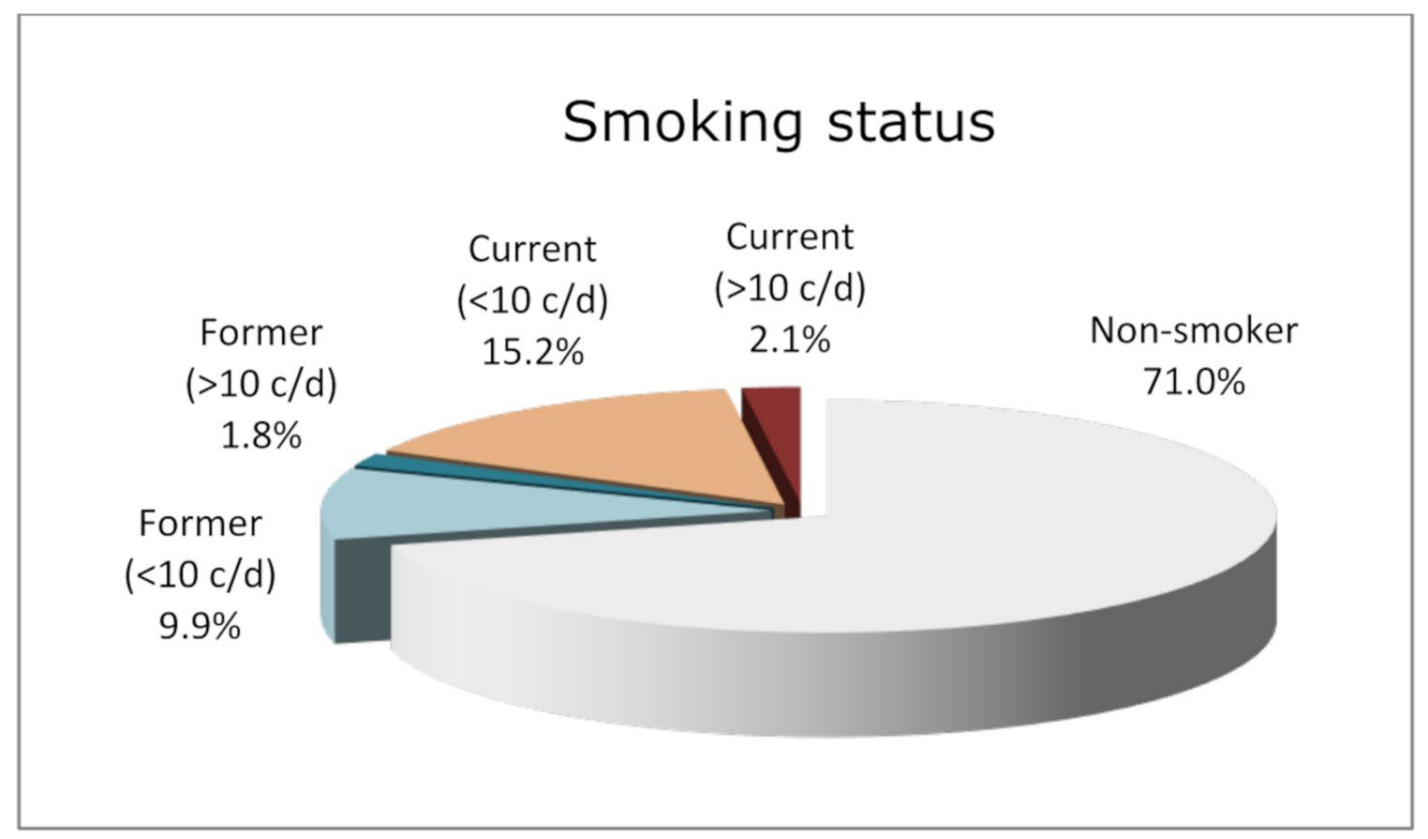
Distribution and stratification of smoking status in the study cohort. The chart illustrates the proportion of non-smokers (71.0%), current smokers (< 10 cigarettes/day: 15.2%; >10 cigarettes/day: 2.1%), and former smokers (< 10 cigarettes/day: 9.9%; >10 cigarettes/day: 1.8%) among participants at baseline

Approximately 14.5% of patients had diabetes. For smoking status, patients were categorized into non-smokers (71%), former light smokers (9.9%), former heavy smokers (1.8%), current light smokers (15.2%), and current heavy smokers (2.1%). Since the number of heavy smokers in both groups was very low, light and heavy smokers were grouped for statistical analysis, making the grouping non-smokers (71%), former light smokers (11.7%), and current smokers (17.3%) (Fig. 1). On average, patients underwent 1.30 ± 0.65 SPT visits per year.

### Analysis of the number of lost teeth due to periodontitis

At baseline (T0), the mean number of present teeth was 24.5 ± 4.2, which decreased to 23.7 ± 5.2 at follow-up (T1). The mean number of teeth lost due to periodontitis during the follow-up period of 19.4 ± 6.9 years was only 0.89 ± 1.87 per patient.

The simple regression analysis (Table 2) revealed that patients with diabetes (OR = 2.41; *p* = 0.013), current smokers (OR = 2.13, *p* = 0.25), Grades B and C (OR = 3.31; *p* = 0.001), and Stages III-IV periodontitis (OR = 8.67; *p* = 0.001) have all shown an increased rate of annual TLP (Table 3).

**Table 2:** Illustrates annual tooth loss and severity of tooth loss due to periodontal diseases (TLP) by independent factors and covariates. Results of simple binary logistic regression (OR and 95%CI, p-value) with GEE model estimation

Annual Tooth Loss	Beta	95% CI	*p*-value
GENDER			
Male	0		
Female	0.001	-0.010 ± 0.012	0.863
AGE	0.0005	0.0001 ± 0.0009	0.018*
DIABETES			
No	0		
Yes	0.017	0.002 ± 0.031	0.031*
SMOKING			0.068
Non-smoker	0		
Former	-0.004	-0.020 ± 0.012	0.601
Current	0.016	0.001 ± 0.030	0.032*
STAGE			0.022*
1	0		
2	0.011	-0.002 ± 0.024	0.095
3–4	0.029	0.008 ± 0.050	0.007**
GRADE			
A	0		
B-C	0.018	0.006 ± 0.030	0.004**
# PROHY VISITS per year	-0.006	-0.014 ± 0.002	0.121
# POCKETS > = 5 mm at T0	0.0017	0.0003 ± 0.0030	0.019*
# POCKETS > = 6 mm at T0	0.0036	-0.0003 ± 0.0075	0.068

**Table 3:** Illustrates annual tooth loss and severity of tooth loss due to periodontal diseases (TLP) by independent factors and covariates. Results of simple binary logistic regression (OR and 95%CI, p-value) with GEE model Estimation

Severity of Tooth Loss	OR	95% CI	*p*-value
GENDER			
Male	0		
Female	0.98	0.57 ± 1.69	0.940
AGE	1.04	1.02 ± 1.07	< 0.001***
DIABETES			
No	0		
Yes	2.41	1.21 ± 4.80	0.013*
SMOKING			0.066
Non-smoker	0		
Former	0.91	0.37 ± 2.23	0.831
Current	2.13	1.10 ± 4.14	0.025*
STAGE			0.001**
1	0		
2	3.07	1.24 ± 7.58	0.015*
3–4	8.67	2.86 ± 26.2	< 0.001***
GRADE			
A	0		
B-C	3.31	1.87 ± 5.86	< 0.001***
# MAINTENANCE VISITS per year	0.50	0.32 ± 0.79	0.003**
# POCKETS > = 5 mm at T0	1.13	1.05 ± 1.22	0.002**
# POCKETS > = 6 mm at T0	1.29	1.09 ± 1.53	0.004**

**Fig. 2:**
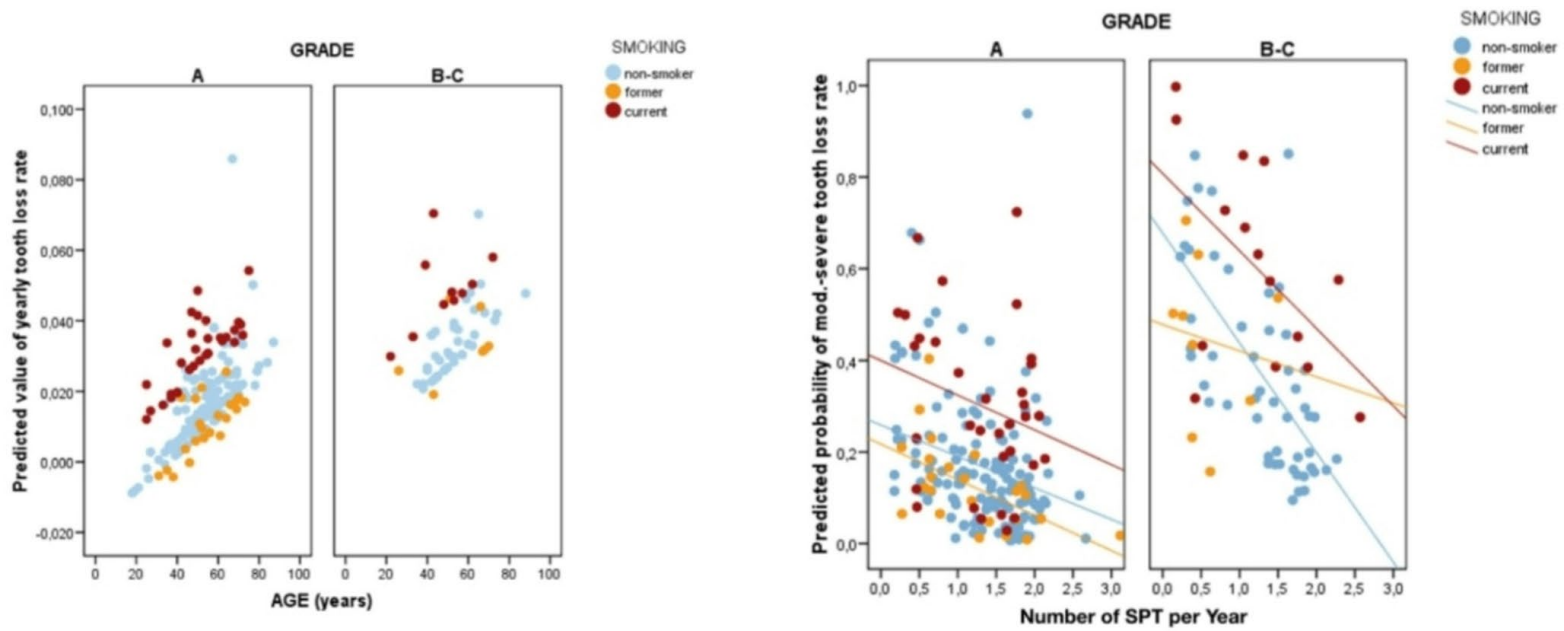
Depicts the annual rate of tooth loss categorized by the model against the three most relevant factors, along with the annual rate of SPT visits

Additionally, the depth of periodontal pockets (≥ 5 mm and ≥ 6 mm) was associated with slightly increased odds of TLP (OR = 1.13 (*p* = 0.002 and 1.29, respectively). While the frequency of non-surgical maintenance was found to have an inverse relationship with the rate of TLP and was statistically significant (OR: 0.50; *p* = 0.003), its protective effect was less evident in mild to moderate cases (Fig. 2).

When multiple regression analyses were performed (Supplementary Table 1), diabetes (OR = 2.86; *p* = 0.014), Current smokers (OR = 1.96, *p* = 0.007), and Grades B-C periodontitis remained significant determinants of TLP rate and had three times higher risk than those with Grade A (OR = 2.78; *p* = 0.016). Furthermore, each additional maintenance visit/ year decreased the risk of TLP by half (54%).

### Factors affecting the number of open pockets

The mean number of PD ≥ 5 mm was 2.01 ± 4.27 at T0 and 2.17 ± 4.82 at T1. The mean annual change in pockets was 0.16 ± 2.70, indicating stability in the number of pockets with PD ≥ 5 mm. The mean number of PD ≥ 6 mm was 0.61 ± 2.01 at T0 and 0.52 ± 1.32 at T1. The mean annual change in PD was − 0.10 ± 1.37, indicating stability in the number of pockets with PD ≥ 6 mm.

Simple Linear Regression Analysis demonstrated that neither diabetes nor current or former smoker status negatively affects mean open pocket reduction in patients under SPT. In fact, the simple regression analysis demonstrated a significant correlation between PD ≥ 6 mm and the annual change in PD but was not clinically relevant. Multiple Regression Analysis (Supplementary Table 3) showed that the only significant predictor of the change in PD ≥ 6 mm was the number of baseline PD ≥ 6 mm.

## Discussion

This study evaluated long-term outcomes of single-visit full-mouth non-surgical therapy by assessing TLP, the prevalence of pockets ≥ 5 mm and ≥ 6 mm, and changes in PD over time. Our analysis shows several factors contributing to TLP, such as diabetes, current smoking, Stages III-IV periodontitis, and Grades B and C. Previous studies support these findings [13, 25, 26]. Despite these risks, the mean TLP was relatively low at 0.9 tooth per patient over approximately 20 years of follow-up. A greater number of residual deep pockets was directly associated with higher odds of tooth loss, while more frequent maintenance visits had a protective effect against TLP. Furthermore, similar to our results, a recent study has discussed that the increased number of teeth with PD ≥ 5 and 6 mm was directly associated with an increased likelihood of TLP [23]. Importantly, baseline PD ≥ 5 mm and ≥ 6 mm were not only predictors of subsequent PD changes but also strong indicators of future tooth loss, consistent with the findings of Eickholz et al. [27] who emphasized the prognostic significance of persistent deep pockets.

Moreover, according to our analysis, non-surgical full-mouth periodontal debridement was found to have a protective effect in mild to moderate cases. Increasing the frequency of SPT visits decreased the risk of severe TLP by half. These findings are consistent with previous studies that found regular SPT visits effectively reduce the risk of further periodontal breakdown and improve clinical outcomes [20, 28–31]. In line with Matuliene et al. (2008), we found that high-risk subgroups especially patients with Stage IV periodontitis accounted for a substantial proportion (62%) of TLP in our cohort, highlighting the importance of tailored interventions for individuals with advanced disease [32]. As noted in (Fig. 2), the trend lines indicate that more frequent SPT visits are associated with a lower rate of TLP, which is more apparent in Grade A than in Grades B-C. However, in line with a plethora of data, current smokers generally showed higher TLP rates than non-smokers and former smokers, regardless of the number of SPT visits [33, 34]. This was particularly evident in higher grades (B-C), where even frequent SPT visits do not reduce the rate of TLP to the levels observed by non-smokers. These findings are in agreement with the results of Graetz et al. (2020) and Pretzl et al. (2018), who reported that structured SPT reduces tooth loss risk, but that smoking remains a strong independent risk factor for adverse periodontal outcomes [35, 36].

Studies involving single-visit full-mouth instrumentation reported significant reductions in the mean number of pockets with a PD greater than or equal to 5 mm and 7 mm after full-mouth disinfection with adjuvant erythritol air-polishing treatment [37, 38]. On the other hand, Jamal Stein’s 2021 study showed that single-visit full-mouth instrumentation had higher reductions in PD and increased pocket closure than quadrant-wise SRP [39]. In line with this, our analysis indicated an almost zero increase in the mean number of pockets ≥ 5 mm and ≥ 6 mm over 20 years. Contrary to TLP, smoking in the present study did not influence the reduction of pockets ≥ 5 mm and ≥ 6 mm. A study by Meulman et al. (2013) compared smokers with severe chronic periodontitis using full-mouth ultrasonic debridement and scaling and root planning and concluded that smokers had a less favorable response than non-smokers, and both treatments were equally effective in terms of clinical outcomes [40]. Similarly, diabetic patients showed a similar tendency towards a reduction in PD, though it did not reach statistical significance.

An RCT by Santos et al. (2009) recorded the mean changes in PD for the full mouth group and at sites with initial PD ≤ 3, 4 to 6, and ≥ 7 mm between baseline and 3 and 6 months, found that there were no significant differences between the treatment groups—full mouth SRP and partial mouth SRP at any time point and hence needs further clarification [41]. While the frequency of SPT was found to have an inverse relationship with the rate of TLP and was statistically significant (OR: 0.50; *p* = 0.003), its protective effect was less evident in milder cases (Fig. 2). This seems to be in line with previous studies [42]. These results, however, should be interpreted with caution. Patient compliance, or the lack thereof, can impact the rate of TLP in patients undergoing periodontal therapy [43–45].

While this study provides valuable insights into the effectiveness of single-visit full-mouth non-surgical therapy, its single-center retrospective design limits generalizability. The patient cohort was drawn from a single academic institution (University of Michigan), which may introduce selection bias due to regional demographic characteristics, standardized treatment protocols, and access to specialized care. For instance, our cohort’s smoking prevalence (18%) and diabetes rates (12%) differ from populations in multi-center studies like Graetz et al. (2020), which reported variations in these risk factors across four German centers (smoking: 9–22%; diabetes: 4–14%) [35]. However, our findings align with Graetz et al.’s (2020) conclusion that structured (SPT) yields low long-term tooth loss rates (0.10–0.15 teeth/patient/year in their study vs. 0.05 in ours). This consistency suggests that single-visit protocols may achieve comparable outcomes to quadrant-wise approaches across diverse settings.

Nevertheless, the homogeneity of our cohort (e.g., strict PiKS inclusion criteria, uniform operator training) may not fully represent real-world scenarios with varied patient adherence, socioeconomic factors, or clinician expertise [35]. Operator experience and professional background can significantly influence the outcomes of non-surgical periodontal therapy. Evidence shows that treatments performed by more experienced clinicians, such as periodontal residents, result in greater reductions in deep pocket depths and a higher percentage of calculus-free root surfaces compared to those performed by dental students or hygienists in training, particularly in moderate and deep pockets [46]. While all operator groups in our study (dental students, hygienists, and periodontics residents) followed a standardized protocol, we acknowledge this variability as a potential confounder.

Despite the growing adoption of comprehensive ultrasonic debridement protocols, there is limited evidence specifically addressing the clinical and microbiological outcomes when the procedure is confined to a maximum of 45 min. Most studies on full-mouth ultrasonic debridement (FMUD) report total treatment times ranging from 45 to 120 min, often depending on disease severity and operator experience [47, 48]. While shorter treatment times offer clear advantages in terms of patient comfort and clinical efficiency, it remains uncertain whether a 45-minute session allows for equally effective disruption of subgingival biofilm and calculus compared to more extended or multi-visit protocols [47, 48].

Some research suggests that the majority of biofilm and endotoxin can be removed with minimal instrumentation time (Quirynen et al., 1995), yet the threshold for optimal clinical improvement—particularly in deep or complex pockets—has not been definitively established for a 45-minute session [49]. Additionally, few studies have systematically evaluated the impact of such a time constraint on host immune-inflammatory responses or long-term periodontal stability. As a result, while the 45-minute approach is promising for its practicality, further research is needed to determine its efficacy relative to longer or staged debridement sessions, especially in patients with advanced periodontitis or multiple risk factors [49].

In our study, periodontal breakdown during SPT (PiKS) was defined as the presence of at least two non-adjacent sites with PD ≥ 5 mm, with or without bleeding on probing. However, recent guidelines and consensus reports, particularly the 2018 classification by the AAP/EFP, emphasize that periodontal breakdown during maintenance should be assessed by the presence of new or progressive clinical attachment loss (CAL) at two or more non-adjacent sites rather than PD alone. Residual deep pockets (≥ 5 mm or ≥ 6 mm) after therapy are recognized as important risk factors for future disease progression and tooth loss, but breakdown is best confirmed by documented CAL loss or radiographic bone loss compared to previous records [24].

## Conclusion

This study highlights the efficacy of a single full-mouth instrumentation in halting disease progression over a long-term follow-up. It demonstrated the significance of diabetes, smoking, periodontitis severity, and initial PD as critical predictors for future TLP. Each additional annual maintenance visit decreased the risk of TLP by half.

It is essential to acknowledge other study limitations. The study’s retrospective nature and tooth-level design may introduce selection bias. The variation among clinical practitioners and the lack of information on specific variables could impact treatment outcomes. Future research could explore the impact of single-visit full-mouth instrumentation compared to quadrant-wise instrumentation in patients with PiKS.

## Electronic supplementary material

Below is the link to the electronic supplementary material.

Supplementary Material 1

## Data Availability

The data used to support the findings of this study are available from the correspondingauthor upon request.

## Abbreviations

SPT: Supportive Periodontal Therapy
TLP: Tooth Loss due to Periodontitis
PiKS: Periodontal Breakdown during SPT
GEE: Generalized Estimation Equations
AAP: American Academy of Periodontology
PMT: Periodontal Maintenance Therapy
SPC: Supportive Periodontal Care
PD: Pocket Depth
APT: Active Periodontal Therapy
SRP: Scaling and Root Planning
BOP: Bleeding on Probing
