# Supplementary material for: Effect of single-visit full-mouth non-surgical therapy and risk factor analysis on long-term periodontal treatment outcomes: A retrospective study
Source: Clin Oral Investig. 2025 Jun 5;29(6):333. doi: 10.1007/s00784-025-06405-2 (PMC12137392; doi:10.1007/s00784-025-06405-2)
Supplement: Supplementary file 1 — Supplementary Material 1 [file 784_2025_6405_MOESM1_ESM.docx]

**SUPPLEMENTAL TABLES**

**Supplemental Table 1** Severity of tooth loss rate by relevant demographic and clinical profile: Results of multiple binary logistic regression (adjusted OR and 95%CI)

| Severity of Tooth loss | OR | 95% CI | p-value |
| --- | --- | --- | --- |
| AGE | 1.05 | 1.02± 1.08 | 0.001** |
| DIABETES |  |  |  |
| No | 0 |  |  |
| Yes | 2.86 | 1.24± 6.59 | 0.014* |
| SMOKING |  |  | 0.008** |
| Non-smoker | 0 |  |  |
| Former | 0.56 | 0.19± 1.62 | 0.285 |
| Current | 1.96 | 1.35± 6.47 | 0.007** |
| STAGE |  |  | 0.202 |
| 1 | 0 |  |  |
| 2 | 2.61 | 0.89± 7.64 | 0.080 |
| 3-4 | 2.18 | 0.45± 10.4 | 0.331 |
| GRADE |  |  |  |
| A | 0 |  |  |
| B-C | 2.78 | 1.21± 6.37 | 0.016* |
| # PROHY VISITS per year | 0.46 | 0.28± 0.77 | 0.003** |
| # POCKETS>=5mm at T0 | 1.09 | 0.96± 1.23 | 0.196 |
| # POCKETS>=6mm at T0 | 1.02 | 0.76± 1.37 | 0.887 |

**Supplemental Table 2** Multiple Regression model shows factors influencing the change in pockets with depth ≥ 5mm.

| of pockets>=5mm | Beta | 95% CI | p-value |
| --- | --- | --- | --- |
| Constant | 0.094 | 0.055± 0.132 | <0.001*** |
| DIABETES |  |  |  |
| No | 0 |  |  |
| Yes | -0.025 | -0.111± 0.062 | 0.573 |
| SMOKING |  |  |  |
| Non-smoker | 0 |  |  |
| Former | -0.128 | -0.220± -0.036 | 0.007** |
| Current | -0.037 | -0.117± 0.043 | 0.369 |
| # POCKETS>=5mm at T0 | -0.030 | -0.041± - 0.019 | <0.001*** |
| # POCKETS>=6mm at T0 | -0.019 | -0.049± 0.012 | 0.225 |

**Supplemental Table 3** Multiple Regression model shows factors influencing the change in pockets with depth ≥ 6mm.

| pockets>=6mm | Beta | 95% CI | p-value |
| --- | --- | --- | --- |
| Constant | -0.024 | -0.074± 0.027 | 0.361 |
| AGE | 0.001 | 0.000± 0.002 | 0.066 |
| # POCKETS>=5mm at T0 | 0.001 | -0.003± 0.006 | 0.516 |
| # POCKETS>=6mm at T0 | -0.046 | -0.059± -0.033 | <0.001*** |
